# Internet Addiction and Sleep Quality Among School Children in South India

**DOI:** 10.7759/cureus.68953

**Published:** 2024-09-08

**Authors:** Lalitha Venugopal, Priyadharsini R, Sathishbabu Murugaiyan, Harini I

**Affiliations:** 1 Physiology, Indira Gandhi Medical College and Research Institute, Puducherry, IND; 2 Pharmacology, Jawaharlal Institute of Postgraduate Medical Education and Research, Puducherry, IND; 3 Biochemistry, Jawaharlal Institute of Postgraduate Medical Education and Research, Karaikal, IND

**Keywords:** adolescent, internet addiction, questionnaire, school children, sleep quality, south india

## Abstract

Background and aim: Today's internet is an unavoidable component of educational sources, the entertainment field, and telecommunication processes. Internet usage is more likely among adolescents and young adults in the form of surfing, chatting, playing games, and fulfilling their social needs. The present study has been planned to assess the effect of internet addiction (IA) on sleep quality in school children.

Methods: This cross-sectional school-based study was conducted among school students in Pondicherry. A total of 350 school students of both genders studying in grades six to 10 and having access to at least one device with internet for more than one year were recruited from government and private schools located in five randomly selected communes in Pondicherry by convenient sampling method. Assessment of IA was done using Young’s Internet Addiction Test (YIAT). The Pittsburgh Sleep Quality Index (PQSI) questionnaire assessed the participants' sleep quality. All the data was tested for normality using the Kolmogorov-Smirnov test. Chi-square and unpaired t-tests were used for analysis.

Results: The average age of the participants is 13 years. 49 (26.1%) are children and 139 (73.9%) are adolescents. The majority of them are females belonging to the adolescent age group (169, 89.4%). Around 17 (19.5%) children and 70 (80.5%) adolescents reported mild IA. Thirteen (59.1%) children and nine (40.9%) adolescents reported moderate IA. There is no significant difference in sleep quality between children and adolescents (4±2.33 vs. 3.62±2.61, p=0.37) but there is a considerable difference in their levels of IA (32.98±21.06 vs. 25.6±15.08, p=0.01).

Conclusion: This study found a significant relationship between IA and poor sleep quality among adolescents. Thus, adolescents, parents, school authorities, and researchers should understand the importance of regulating internet usage and encouraging sleep hygiene. Thus, appropriate measures should be taken to address the effects of IA on sleep quality.

## Introduction

The development of internet services has revolutionized the various facets of modern life. Its advent has been embedded deeply into every individual activity in daily life. Thus, it has become essential to our day-to-day. Though the internet offers many benefits, like access to educational resources, information, and social connectivity, it is also associated with adverse outcomes or potential risks. In India, the proportion of people accessing the internet increased from 17% in 2005 to 53.6% in 2019 [1]. Internet usage in developed countries is high and it is around 86.6% [2]. Today's internet is an unavoidable component of educational sources, the entertainment field, and telecommunication processes. The ability to limit internet usage varies from individual to individual. Limited use of the internet based on time varies with the capability of various individuals affecting autonomic functions [3]. Internet usage is more likely among adolescents and young adults in the form of surfing, chatting, playing games, and fulfilling their social needs. It was found that most of them used the internet only for information gathering. With the increased availability of devices such as tablets, mobile phones, and other devices, adolescents have more access to the internet throughout the day and night. This excessive internet usage is described as internet addiction (IA) [4].

IA has raised an emerging problem among school children. It affects sleep quality and overall well-being. Adolescents with poor self-control are more prone to IA than adults. These may lead to health problems like anxiety and depression [5]. The IA does not merely represent the omnipresence of the internet, but it reflects the role of psychological, social, and environmental factors interplay. The convenience provided by the internet may lead to different escapisms for children due to societal pressure, academic stress, and other anxieties. This causes the children to lose control over their online behaviors and heightened addictive potential. The repercussions of IA are significant challenges and affect physical health, including sleep quality. IA negatively impacts the individual's overall health. It has an adverse effect on sleep quality. IA is reported to be associated with poor disturbances in sleep quality [6,7]. Poor sleep quality is related to physical, mental, and cognitive dysfunctions. Previous studies have shown that the sleep hours per night have decreased in all age groups with IA. Insufficient sleep across the lifespan is associated with the risk of obesity, hypertension, and type 2 diabetes [8,9].

There should be a collaborative approach by healthcare providers, parents, and educators to address IA and mitigate its adverse effects. These efforts are crucial, ensuring the children reap the benefits of the digital age without compromising their growth and development. Family should foster a healthy relationship with digital technology. Although it is a growing concern, most studies on IA and sleep quality have been conducted only in adults. There is a lack of research on IA and sleep quality among the pediatric population. Therefore, in the present study, we have planned to assess the effect of IA on sleep quality in school children.

## Materials and methods

This cross-sectional school-based study was conducted among the school students in Pondicherry. After the approval from the Institute Scientific Committee and Ethics Committee for Human Studies (IEC registration number:454/IEC-37/PP-5/2023), a total of 350 school students (seven to 12 years of age is considered as children and 13 to 18 years of age is considered as adolescents) of both genders studying in grades six to 10 (age group of 11-16 years) having access to at least one device with internet for more than one year were recruited from government and private schools located in five randomly selected communes in Pondicherry (Puducherry, Oulgaret, Villianur, Nettapakkam, and Ariyankuppam) were approached. However, only 188 students completed the questionnaire. Students with a known history of any medical, surgical, psychological, or sleep disorders were excluded from the study. Assuming the prevalence of adolescents having access to at least one device with internet for more than one year as 50%, alpha error of 5% (95% confidence level), and 8% absolute precision, the conditioned sample size calculated was 350. The sample size was calculated using Open-epi software version 3. A convenient sampling method was used. Permission from the schools was obtained from the Directorate of Education and the respective schools' Head of the Institution (Principal) before the onset of the study. The objective, procedure, and confidentiality of the survey were explained, and a written informed consent and assent form was obtained from the participants and parents before the onset of the study. Internet usage and sleep quality were assessed using questionnaires. Prior copyright permission was obtained from the developers.

Information regarding age, gender, and the studying class was gathered from every participant. Assessment of IA was done using Young’s Internet Addiction Test (YIAT). YIAT-20 is a self-administered, 20-item questionnaire answered on a 5-point Likert scale (0 - does not apply, 1 - rarely, 2 - occasionally, 3 - frequent, 4 - often, 5 - always). Total IA scores are calculated by adding 20 items with possible scores ranging from 0 to 100. Based on the scoring, subjects were classified into normal users (≤20), mild (21-49), moderate (50-79), and severe (80-100) IA groups (score ≤20 - IA absent; score >20 - IA present) [4,5]. The Pittsburgh Sleep Quality Index (PQSI) questionnaire assessed the participants' sleep quality. PSQI is a 19-item tool that evaluates sleep quality for a month. It examines the seven components of sleep: sleep quality, latency, sleep duration, sleep efficiency, details of using sleeping pills, and daytime dysfunction. PSQI score ranges from 0 to 21. A score above or equal to 5 was considered poor sleep quality.

Statistical analysis

All the data was tested for normality using the Kolmogorov-Smirnov test. Normally distributed data was expressed in Mean ± standard deviation (SD). Categorical data like level of IA, IA status (present/absent), and sleep quality (good, poor) will be expressed in frequencies. Analysis of data was done using Graph pad InStat (Version 3, USA) software. A p-value of <0.05 was considered statistically significant.

## Results

This cross-sectional school-based study was conducted among a total of 188 school students of both genders studying in grades six to 10 (age group of 11-16 years) having access to at least one device with internet for more than one year recruited from government and private schools located in five randomly selected communes in Pondicherry (Puducherry, Oulgaret, Villianur, Nettapakkam, and Ariyankuppam) in Pondicherry. Out of the 350 questionnaires, nearly 188 participants completed the questionnaire, and the response rate was around 51.4%. Table 1 shows the demographic characteristics of study participants. It summarizes the age distribution and gender composition of the study participants. The average age of the participants is 13 years. The age range is from 11 to 16 years. 49 (26.1%) are children and 139 (73.9%) are adolescents. The majority of them are adolescents and females (168, 89.4%).

**Table 1:** Demographic characteristics of study participants (N=188) ^#^Parameters are expressed as mean ± standard deviation.

Variable	N (%)
Age (years)^#^ (range)	13.21±0.96 (11,16)
Children	49 (26.1)
Adolescent	139 (73.9)
Male	20 (10.6)
Female	168 (89.4)

Table 2 shows the sleep quality and mild IA among study participants. Most have good sleep quality (132, 70.2%) and 87 (46.3%) had mild IA.

**Table 2:** IA and sleep quality characteristics of study participants (N=188) ^#^Parameters are expressed as mean ± standard deviation. PSQI, Pittsburgh Sleep Quality Index, a measure of sleep quality; IAT, internet addiction test, a measure of IA; IA, internet addiction

Variable	N (%)
PSQI^#^ (range)	3.72±2.54 (0,15)
Good sleep	132 (70.2)
Poor sleep	56 (29.8)
IAT^#^ (range)	27.5±17.08 (0,72)
IAT	-
No addiction	79 (42)
Mild	87 (46.3)
Moderate	22 (11.7)
Severe	0

Table 3 compares sleep quality characteristics among 188 study participants who are categorized into different groups based on age and gender. Nearly 15 (26.8%) children reported poor sleep quality, while 41 adolescents (73.2%) reported poor sleep quality. The P-value for this comparison is 0.51, indicating no significant difference in sleep quality between children and adolescents. Ten males (7.6%) reported good sleep quality, while 122 females (92.4%) reported the same. On the contrary, 10 (17.9%) males reported poor sleep quality, while 46 (82.1%) females reported poor sleep quality. The Chi-square test was used to compare the good and poor sleep quality proportions between the groups. The P-value for this comparison is 0.07, suggesting a trend toward significance.

**Table 3:** Comparison of sleep quality characteristics of study participants (N=188) P<0.05 was considered significant. The Chi-square test was used for comparison.

Variable	Good sleep, N (%)	Poor sleep, N (%)	P-value
Children	34 (25.8)	15 (26.8)	0.51
Adolescent	139 (73.9)	41 (73.2)	-
Male	10 (7.6)	10 (17.9)	0.07
Female	122 (92.4)	46 (82.1)	-

Table 4 compares the characteristics of IA among 188 study participants by age and gender. Seventeen (19.5%) children and 70 (80.5%) adolescents reported mild IA. Thirteen (59.1%) children and nine (40.9%) adolescents reported moderate IA. The p-value for this comparison is 0.001, indicating a very significant difference in IA levels between children and adolescents. Five (6.3%) males and 74 (93.7%) females reported no IA. Eight (9.2%) males and 79 (90.8%) females reported mild IA. Seventeen (19.5%) males and 15 (68.2%) females reported moderate IA. The P-value for this comparison is 0.002, indicating a very significant difference in IA levels between males and females.

**Table 4:** Comparison of IAT characteristics of study participants (N=188) p<0.05 was considered significant, p<0.01 was considered very significant, and p<0.001 was considered extremely significant. The Chi-square test was used for comparison. IAT, internet addiction test

Variable	No addiction, N (%)	Mild addiction, N (%)	Moderate addiction, N (%)	P-value
Children	19 (24.1)	17 (19.5)	13 (59.1)	0.001
Adolescent	60 (75.9)	70 (80.5)	9 (40.9)	-
Male	5 (6.3)	8 (9.2)	17 (19.5)	0.002
Female	74 (93.7)	79 (90.8)	15 (68.2)	-

Table 5 and Table 6 show significant differences in IA and sleep quality among different age groups and genders. Table 5 compares the sleep quality of participants based on their levels of IA. Around 63 (47.7%) participants with good sleep quality had no addiction, while 58 (43.9%) and 11 (8.3%) had mild and moderate addictions, respectively. Similarly, 16 (28.6%) participants with poor sleep quality had no addiction, while 29 (51.8%) and 11 (19.6%) had mild and moderate addictions, respectively. The P-value is 0.02, indicating a significant difference in sleep quality based on IA.

**Table 5:** Comparison of IA and sleep quality characteristics of study participants (N=188) p<0.05 was considered significant. The Chi-square test was used for comparison. IA, internet addiction

Variable	Good sleep, N (%)	Poor sleep, N (%)	P-value
No addiction	63 (47.7)	16 (28.6)	0.02
Mild addiction	58 (43.9)	29 (51.8)	-
Moderate addiction	11 (8.3)	11 (19.6)	-

**Table 6:** Comparison of IA and sleep quality scores of study participants by age and gender (N=188) p<0.05 was considered significant. Parameters are expressed as mean ± standard deviation. An unpaired t-test was used for comparison. PSQI, Pittsburgh Sleep Quality Index, a measure of sleep quality; IAT, internet addiction test, a measure of IA; IA, internet addiction

Variable	Children	Adolescent	P-value		Male		Female		P-value
PSQI	4±2.33	3.62±2.61		0.37		4.65±3.77		3.61±2.34	0.08
IAT	32.98±21.06	25.6±15.08		0.01		40.25±20.7		25.98±16	0.000

Table 6 highlights the comparison that while there is no significant difference in sleep quality between children and adolescents, there is a considerable difference in their levels of IA. The average PSQI score is 4, with a standard deviation of 2.33 in children. Still, in adolescents, the average PSQI score is 3.62, with a standard deviation of 2.61, indicating no significant difference in sleep quality between children and adolescents. Similarly, the average IAT score is 32.98, with a standard deviation of 21.06 in children. However, in adolescents, the average IAT score is 25.6, with a standard deviation of 15.08, indicating a significant difference in IA levels between children and adolescents.

## Discussion

IA has extended beyond a lot, affecting the immediate behavioral and psychological domains. It has become evident that internet usage affects various aspects of physical health, most notably sleep quality [10,11].

Sleep is a critical component in a child's development. It influences cognitive functioning, emotional regulation, physical growth, and well-being. It plays a vital role in enhancing school performance, memory consolidation, and the ability to handle daily stressors. However, IA can disrupt sleep patterns through various mechanisms. They may prolong screen time, exposure to blue light, and increased arousal due to engaging in online content [12]. Thus, the disruption of sleep can lead to various adverse outcomes such as daytime sleepiness, impaired academic performance, mood disturbances, and an increased risk of developing chronic health conditions [13]. Additionally, the stimulating nature of internet activities, such as gaming, social networking, and watching videos, can increase mental alertness and make it difficult for children to unwind and fall asleep. Some studies have proven that excessive internet use, especially before bedtime, can significantly impair sleep quality among school children [14]. The emission of blue light by screens can interfere with melatonin production. This hormone plays an essential role in maintaining the circadian rhythm. It regulates sleep-wake cycles and delays sleep onset and duration [15].

This study found a significant difference in IA levels between children and adolescents. The adolescents were showing higher levels of mild and moderate addiction (P-value=0.001). The findings from this study align with and expand upon existing research on IA and sleep quality among different age groups and genders [16,17]. Similar results have been reported in previous research [18]. For instance, a study by Throuvala et al. (2014) indicated that adolescents are more prone to IA compared to younger children as adolescents have increased access to digital devices and social media platforms [19]. Similarly, other studies have shown the same results [18,20]. The present study observed a significant difference in IA levels between males and females, with males showing higher levels of moderate addiction (P-value=0.002). This finding is consistent with research by Durkee et al. (2012), which found that males are generally more susceptible to IA than females, possibly due to different online activities and usage patterns [21]. Additionally, Wang et al. found that females have been found to report poorer sleep quality compared to males, potentially due to hormonal differences and higher levels of stress and anxiety [18,22].

The study highlighted a significant relationship between IA and poor sleep quality (P-value=0.02). Participants with higher levels of IA reported poorer sleep quality. This is supported by research from Lam (2014), which demonstrated that excessive internet use is associated with sleep disturbances and poorer sleep quality among adolescents [23]. The mechanisms by which excessive internet usage affects include increased screen time leading to delayed sleep onset and reduced sleep duration. No significant difference in sleep quality was found between children and adolescents (P-value=0.37) or between males and females (P-value=0.08). Contrastingly, some studies, such as those by Gradisar et al. (2011), have reported that adolescents generally experience poorer sleep quality than younger children due to biological changes and increased academic and social pressures [22,24].

The strengths of this study lie in its comprehensive data collection from various regions of Puducherry, use of validated instruments as described in previous studies, identification of trends in the IA, and focus on adolescents. These strengths provide valuable insights into the relationship between IA and sleep quality among different age groups and genders.

Our study has a few limitations. Our study data is self-reported data. We relied on self-reported measures for both IA and sleep quality. Hence, self-reported data can be subject to biases such as social desirability bias and recall bias. This may affect the accuracy of the results. The cross-sectional nature of the study design captures data at a single point in time. This limits the ability to infer a causal relationship between IA and sleep quality. Therefore, longitudinal studies would be needed to establish causal relationships. In addition, we did not consider the confounding factors that could influence IA and sleep quality, such as socioeconomic status, culture, mental health conditions, and other lifestyle factors, which may substantially affect the study because of logistic feasibility. Hence, further studies of a larger population are required. The study had a higher proportion of adolescents and females, which might skew the results. A more balanced distribution of age and gender could provide a more comprehensive understanding of the relationships being studied. Larger sample sizes could provide more robust and generalizable results. The study does not account for temporal variations in internet usage and sleep patterns, such as between weekdays and weekends or seasonal variations.

Based on the findings of this study, several recommendations can be made to address IA and improve sleep quality among different age groups and genders. Appropriate educational programs should be implemented in schools. This may raise awareness about the risks of excessive internet use and its impact on sleep quality. These programs can include strategies for managing screen time and promoting healthy sleep habits. Parents should be provided with educational resources like setting time limits on screen time and appropriate continued educational programs like workshops to help them understand the importance of regulating their children’s internet use and encouraging good sleep hygiene. Digital detox periods to reduce dependency on internet usage, such as meditation and other relaxation techniques, should be promoted. Other methods, such as counseling, mental health support, establishing peer support groups, and policy decisions on digital literacy, should be focused on. By implementing these recommendations, it is possible to mitigate the negative impacts of IA on sleep quality and promote healthier lifestyles among children, adolescents, and adults. These strategies can help create a balanced approach to internet use and improve overall well-being.

## Conclusions

Our study found a significant relationship between IA and poor sleep quality among adolescents. Thus, adolescents, parents, school authorities, and researchers should understand the importance of regulating internet usage and encouraging sleep hygiene. This highlights the importance of mitigating the impacts of IA on sleep quality. Future studies should investigate the root causes of patterns of excessive internet usage, and policy decisions on digital literacy should be recommended.

## References

[1] Karki K, Singh DR, Maharjan D, K C S, Shrestha S, Thapa DK (2021). Internet addiction and sleep quality among adolescents in a peri-urban setting in Nepal: a cross-sectional school-based survey. PLoS One.

[2] (2024). News ITU. Measuring digital development: Facts & figures. https://www.itu.int/hub/2020/05/measuring-digital-development-facts-figures-2019/.

[3] Nayak A, Saranya K, Fredrick J, Madumathy R, Subramanian SK (2024). Assessment of burden of internet addiction and its association with quality of sleep and cardiovascular autonomic function in undergraduate medical students. Clin Epidemiol Glob Health.

[4] Prashanthi B (2017). Internet usage pattern of adolescents. IOSR JHSS.

[5] Orsal O, Orsal O, Unsal A, Ozalp SS (2013). Evaluation of internet addiction and depression among university students. Procedia Soc Behav Sci.

[6] Wolniczak I, Cáceres-DelAguila JA, Palma-Ardiles G (2013). Association between Facebook dependence and poor sleep quality: a study in a sample of undergraduate students in Peru. PLoS One.

[7] Khayat MA, Qari MH, Almutairi BS (2018). Sleep quality and Internet addiction level among university students. Egypt J Hosp Med.

[8] Phillips SR, Johnson AH, Shirey MR, Rice M (2020). Sleep quality in school-aged children: a concept analysis. J Pediatr Nurs.

[9] Benjamin EJ, Muntner P, Alonso A (2019). Heart disease and Stroke Statistics-2019 update: a report from the American Heart Association. Circulation.

[10] Çelebioğlu A, Aytekin Özdemir A, Küçükoğlu S, Ayran G (2020). The effect of internet addiction on sleep quality in adolescents. J Child Adolesc Psychiatr Nurs.

[11] Guclu Y, Guclu OA, Demirci H (2024). Relationships between internet addiction, smartphone addiction, sleep quality, and academic performance among high-school students. Rev Assoc Med Bras (1992).

[12] Eliacik K, Bolat N, Koçyiğit C (2016). Internet addiction, sleep and health-related life quality among obese individuals: a comparison study of the growing problems in adolescent health. Eat Weight Disord.

[13] Nakshine VS, Thute P, Khatib MN, Sarkar B (2022). Increased screen time as a cause of declining physical, psychological health, and sleep patterns: a literary review. Cureus.

[14] Sakamoto N, Kabaya K, Nakayama M (2022). Sleep problems, sleep duration, and use of digital devices among primary school students in Japan. BMC Public Health.

[15] Sakamoto N, Gozal D, Smith DL (2017). Sleep duration, snoring prevalence, obesity, and behavioral problems in a large cohort of primary school students in Japan. Sleep.

[16] Wolde A, Aydiko A (2022). Sleep quality among adolescents and its relation to inhalant, khat, and internet use, and physical illness: a community-based exploratory cross-sectional study. Glob Pediatr Health.

[17] Feher A, Fejes E, Kapus K (2023). The association of problematic usage of the internet with burnout, depression, insomnia, and quality of life among Hungarian high school students. Front Public Health.

[18] Wang Y, Zhao Y, Liu L, Chen Y, Ai D, Yao Y, Jin Y (2020). The current situation of Internet addiction and its impact on sleep quality and self-injury behavior in Chinese medical students. Psychiatry Investig.

[19] Throuvala MA, Griffiths MD, Rennoldson M, Kuss DJ (2019). School-based prevention for adolescent internet addiction: prevention is the key. A systematic literature review. Curr Neuropharmacol.

[20] Kokka I, Mourikis I, Nicolaides NC, Darviri C, Chrousos GP, Kanaka-Gantenbein C, Bacopoulou F (2021). Exploring the effects of problematic Internet use on adolescent sleep: a systematic review. Int J Environ Res Public Health.

[21] Durkee T, Kaess M, Carli V (2012). Prevalence of pathological internet use among adolescents in Europe: demographic and social factors. Addiction.

[22] Acikgoz A, Acikgoz B, Acikgoz O (2022). The effect of internet addiction and smartphone addiction on sleep quality among Turkish adolescents. PeerJ.

[23] Lam LT (2014). Risk factors of Internet addiction and the health effect of internet addiction on adolescents: a systematic review of longitudinal and prospective studies. Curr Psychiatry Rep.

[24] Gradisar M, Gardner G, Dohnt H (2011). Recent worldwide sleep patterns and problems during adolescence: a review and meta-analysis of age, region, and sleep. Sleep Med.

